# The role of demographic history and selection in shaping genetic diversity of the Galápagos penguin (*Spheniscus mendiculus*)

**DOI:** 10.1371/journal.pone.0226439

**Published:** 2020-01-07

**Authors:** Gabriella Arauco-Shapiro, Katelyn I. Schumacher, Dee Boersma, Juan L. Bouzat

**Affiliations:** 1 Department of Biological Sciences, Bowling Green State University, Bowling Green, Ohio, United States of America; 2 Center for Ecosystem Sentinels and Department of Biology, University of Washington, Seattle, Washington, United States of America; University of Iceland, ICELAND

## Abstract

Although many studies have documented the effects of demographic bottlenecks on the genetic diversity of natural populations, there is conflicting evidence of the roles that genetic drift and selection may play in driving changes in genetic variation at adaptive loci. We analyzed genetic variation at microsatellite and mitochondrial loci in conjunction with an adaptive MHC class II locus in the Galápagos penguin (*Spheniscus mendiculus*), a species that has undergone serial demographic bottlenecks associated with El Niño events through its evolutionary history. We compared levels of variation in the Galápagos penguin to those of its congener, the Magellanic penguin (*Spheniscus magellanicus*), which has consistently maintained a large population size and thus was used as a non-bottlenecked control. The comparison of neutral and adaptive markers in these two demographically distinct species allowed assessment of the potential role of balancing selection in maintaining levels of MHC variation during bottleneck events. Our analysis suggests that the lack of genetic diversity at both neutral and adaptive loci in the Galápagos penguin likely resulted from its restricted range, relatively low abundance, and history of demographic bottlenecks. The Galápagos penguin revealed two MHC alleles, one mitochondrial haplotype, and six alleles across five microsatellite loci, which represents only a small fraction of the diversity detected in Magellanic penguins. Despite the decreased genetic diversity in the Galápagos penguin, results revealed signals of balancing selection at the MHC, which suggest that selection can mitigate some of the effects of genetic drift during bottleneck events. Although Galápagos penguin populations have persisted for a long time, increased frequency of El Niño events due to global climate change, as well as the low diversity exhibited at immunological loci, may put this species at further risk of extinction.

## Introduction

Demographic bottlenecks are major contributors to reduced genetic diversity in natural populations. Such reductions may have significant consequences for the long-term persistence of populations, as these may result in decreased fitness (e.g., through inbreeding depression), lowering long-term evolutionary potential, and thus, increase extinction probabilities [1, 2]. According to bottleneck theory, both heterozygosity and number of alleles at neutral loci tend to decrease after significant reductions in population size [3, 4]. However, since low frequency alleles are commonly lost during bottleneck events, allelic diversity is expected to decrease at a faster rate than heterozygosity [4]. Similarly, low levels of genetic diversity may be expected in species with restricted distributions and relative low abundances over evolutionary time [5, 6, 7, 8].

Adaptive loci tend to exhibit genetic diversity patterns similar to neutral loci, with changes in allele frequencies in small populations dominated by genetic drift. However, adaptive loci may be subjected to strong selective pressures affecting the dynamics of their allele frequencies, which may depart from the stochastic expectations of drift [4]. An important set of adaptive genes is the major histocompatibility complex (MHC), which code for cell surface glycoproteins that recognize and present antigens to T cells, thereby initializing the adaptive immune response in vertebrates [9, 10]. MHC genes are usually extremely polymorphic, with many alleles and high nucleotide diversity [11, 12]. In particular, exon 2 of MHC class II β genes is highly polymorphic because it codes for the peptide-binding region involved in the immune response [13, 14]. MHC polymorphisms are generated by mutation and recombination, and maintained by multiple processes including balancing selection, gene conversion, disassortative mating, and/or maternal-fetal interactions [9, 13, 15, 16, 17]. It is thought that the coevolution of MHC and pathogens represents the main mechanism maintaining MHC diversity through the action of balancing selection [18], by means of overdominance [19, 20], negative frequency-dependent selection [21, 22], or diversifying selection [23]. Thus, strong selection acting at MHC loci during bottleneck events could potentially counter the effect of genetic drift, thereby preventing the loss or fixation of alleles [9, 20, 24].

There is conflicting evidence of the potential role of selection in maintaining genetic diversity in the face of strong genetic drift. Most studies of MHC variation suggest that selection at MHC loci may be outweighed by genetic drift, particularly in small and bottlenecked populations (e.g., [25, 26, 27, 28]). However, under strong balancing selection, one would expect higher levels of MHC diversity than neutral variation to be maintained after a bottleneck event [29]. Evidence for strong balancing selection maintaining MHC diversity includes finding a larger number of alleles at MHC compared to neutral loci and a relatively high divergence of MHC alleles [30]. Such expectations of balancing selection during bottlenecks are not always applicable since natural selection may change patterns of diversity occasionally (e.g., due to the sporadic presence of pathogens) rather than continuously [31]. Consequently, adaptive loci may actually experience near-neutral evolution.

The potential effects of selection are also related to the effective size (N_e_) of populations, in that N_e_ affects selection coefficients and thus allows for greater changes in the frequencies of advantageous alleles due to the action of natural selection [32, 33]. Since demographic bottlenecks result in a significant decrease in N_e_, genetic drift would tend to outweigh the effect of balancing selection on MHC variation [34, 35]. While most studies suggest that MHC loci become effectively neutral during population bottlenecks (e.g., [12, 25, 26, 28]), some studies have found that balancing selection can outweigh genetic drift and produce recent signatures of selection [36, 37, 38, 39, 40, 41]. Thus, there is still considerable debate about the relative roles that natural selection and genetic drift may play in shaping genetic diversity during population bottlenecks [2, 42, 43, 44, 45, 46].

Penguin species in the genus *Spheniscus* provide a good study system for assessing the potential effects of small population size on the genetic diversity of natural populations. The endangered Galápagos penguin (*Spheniscus mendiculus*), a species endemic to the Galápagos Islands, has a restricted distribution within 400 kilometers off the coasts of Fernandina and Isabela Islands, where the Cromwell Current upwells and brings the most productive water around the islands [47, 48, 49]. An extremely influential factor on Galápagos penguin demography is the history of El Niño-Southern Oscillation (ENSO) events, which limit the upwelling and oceanic productivity around the islands and have shaped the breeding biology of this species [47]. Unlike other penguins, the Galapagos penguin only breeds when conditions are favorable and deserts eggs or young when upwelling fails [50]. The ENSO events have a long history, but the most recent recordings of severe bottlenecks affecting Galápagos penguins are those of 1982–83 and 1997–98, which may have reduced the population by approximately 77 and 65 percent, respectively [48, 51, 52, 53]. The frequency of the ENSO events hampers recovery of the population, with growing concerns that both the frequency and severity of these events are increasing in recent history due to global warming [53, 54, 55]. The population in 2009 was likely between 1,800 and 4,700 individuals, with population estimates varying from a high of 10,000 individuals in 1971 to a low of 700 in 1983 [49]. Their breeding biology reflects their long history associated with their relatively low abundance and the unpredictable environment of the Galápagos Islands [47, 56]. In contrast, the Magellanic penguin (*S*. *magellanicus*) has approximately 1.1 to 1.6 million breeding pairs that nest along the eastern and western coasts of South America, from Cape Horn to 42°S, as well as the Malvinas/Falkland Islands [49, 57]. The foraging area of Magellanic penguins is less affected by El Niño events, and includes a large continental shelf that provides substantial food for this species during breeding and migration [58, 59]. Furthermore, the species has been through a population expansion since the last glacial maximum [60]. Thus, Magellanic penguins have maintained much larger populations over their evolutionary history, with breeding colony sizes reaching 200,000 to 400,000 pairs [59, 61, 62].

The contrasting demographic histories of the Galápagos and Magellanic penguins may have significant effects on their respective genetic diversity, as larger populations can maintain higher variation [63] and demographic bottlenecks can lead to significant decreases in genetic diversity [2, 64]. Two independent studies have previously documented bottleneck effects on neutral and adaptive genetic variation of Galápagos penguins. Akst et al. [65] compared neutral genetic diversity between 46 Galápagos and 46 Magellanic penguins using five microsatellite markers, and found much lower diversity in the Galápagos population, both in terms of having fewer alleles per locus and a significant reduction in heterozygosity (only 3% compared to 46% in the Magellanic penguin). Bollmer et al. [11] analyzed adaptive genetic diversity at the MHC class II DRβ1 exon 2 and found only three alleles across 30 Galápagos penguin samples, yet limited sampling of Magellanic penguins (only one individual was analyzed for comparison) prevented any major inference regarding levels of variation at this locus. Both studies suggested that genetic drift, due to founder effects and subsequent bottlenecks, was most likely responsible for the reduced genetic diversity in Galápagos penguins [11, 65]. However, it is still unclear whether natural selection has acted or is currently acting to preserve the remaining diversity, particularly at MHC loci.

A thorough assessment of the genetic variation at both adaptive and neutral loci in Galápagos and Magellanic penguins would build upon the results of Akst et al. [65] and Bollmer et al. [11], who used different markers on distinct sample sets. Only studies that use both adaptive and non-adaptive loci would be able to assess genome-wide effects of genetic drift in species subjected to bottleneck events, potentially disentangling signatures of genetic drift and selection. In addition, genetic inferences from bottlenecked populations are only possible when these are compared to proper “control” populations (i.e., non-bottlenecked reference populations) selected in a manner that compensates for potential discrepancies in geographic range and sample sizes.

In this study, we assessed the genetic diversity of Galápagos and Magellanic penguins to evaluate the potential roles of genetic drift and selection on variation at both neutral (mitochondrial and microsatellite) and adaptive (MHC) loci. We tested the overall hypothesis that the relatively low abundance and restricted distribution of Galápagos penguins, in conjunction with a demographic history of serial bottlenecks associated with ENSO events, led to an overall reduction of genetic diversity at both neutral and adaptive loci due to the effects of genetic drift operating in small populations. The Magellanic penguin has not undergone such population size reductions, and as a closely related species can be used as a non-bottlenecked “control,” which should maintain higher genetic variation overall. The joint analyses of neutral and adaptive loci in these two demographically distinct species allowed for the investigation of the relative roles of drift and selection in maintaining diversity at the MHC in a species with comparatively low abundance and a history of demographic bottlenecks. Concordant patterns of neutral and MHC variation would be indicative of the predominant role of genetic drift, while selection effects in the serially bottlenecked Galápagos penguin would result in higher MHC polymorphisms than those expected under neutral diversity. Furthermore, analyses of sequence divergence and allele frequencies at the MHC would enable differentiating between long-term evolutionary versus contemporary signals of selection. Understanding the evolutionary processes affecting genetic diversity in populations that have undergone serial demographic bottlenecks may reveal significant insights into the long-term survivability of the Galápagos penguin, especially since genetic diversity may correlate to adaptability [66, 67], survival [68, 69, 70], and resistance to diseases [71].

## Materials and methods

### Ethics statement

Blood sampling of Magellanic penguins was conducted under protocols approved by the local authorities of Argentina (Division of Fauna and Flora and Department of Tourism of Argentina), as well as USDA-APHIS importation permit #42579 to Robert C. Faucett (University of Washington Burke Museum of Natural History). Sampling of Galápagos penguin blood was conducted with permits from the Galápagos National Park Service, Ecuador, to Dee Boersma.

### Sample collection

**Fig 1:**
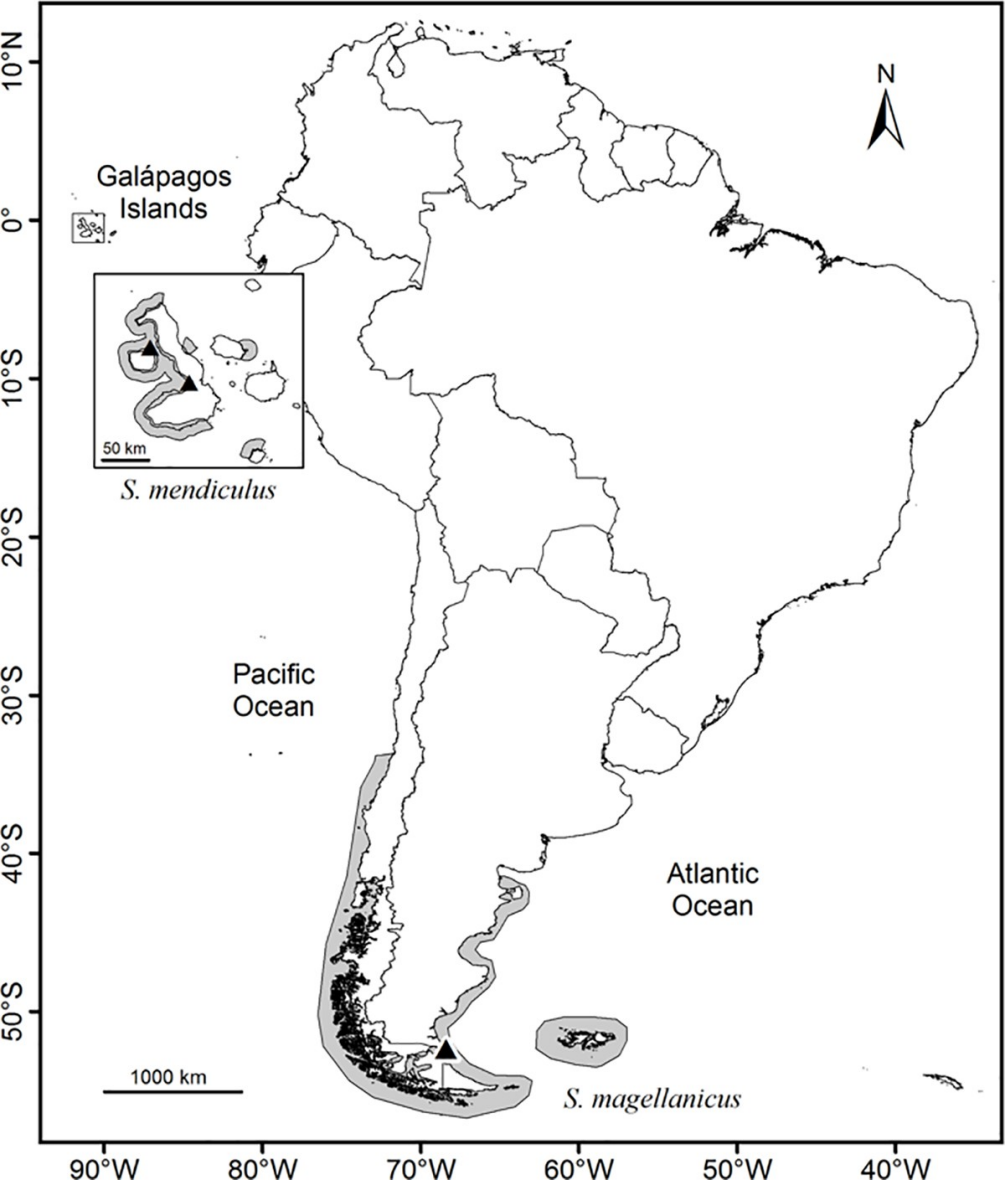
Map of South America overlaid with *Spheniscus magellanicus* (Magellanic penguin) and *S*. *mendiculus* (Galápagos penguin) breeding ranges (gray shading) and sampling localities for this study (triangles). The wide-ranging Magellanic penguin was sampled (N = 26) from a large breeding colony located at the southeastern tip of continental Argentina (Cabo Vírgenes). Endemic to the Galápagos Islands, the Galápagos penguin was sampled (N = 38) from Elizabeth Bay on Isabela Island, the largest island in the archipelago, as well as from Punta Espinoza on the nearby Fernandina Island. Spatial data for breeding ranges of the penguins was extracted from the global dataset of avian distribution maps [77].

Blood samples (~100–200 μl) from penguins were collected by puncture of the brachial or foot veins and stored in Queen’s lysis buffer (0.01 M Tris, 0.01 M NaCl, 0.01 M EDTA, and 1% *n*-lauroylsarcosine, pH 7.5; [72]). Species sampling included 26 Magellanic penguins from Cabo Vírgenes, a large breeding colony located in southern Argentina (52°20’S, 68°21’W), and 38 Galápagos penguins from the Galápagos Islands (Fig 1). The Cabo Vírgenes samples (collected in 2001) were selected with the assumption that they would be representative of the genetic variation potentially found in other Magellanic colonies, as this colony is rather large with about 89,200 breeding pairs (1994 estimate; [73]). The localized range of the Cabo Vírgenes colony prevents the potential detection of increased diversity due to geographic differentiation of colonies from disparate locations, and thus provides a more conservative control for comparison, since the inclusion of other colonies may increase estimates of genetic diversity due to geographic structuring [74], in support of our hypothesis. That said, previous studies [60, 75] have shown high levels of gene flow among Magellanic penguin colonies. Therefore, the sampling of Cabo Vírgenes may also capture genetic diversity from neighboring colonies. Galápagos penguin samples were collected in 1997–98 from two islands (Fernandina and Isabela), to ensure a better representation of the species’ genetic diversity throughout the Galápagos archipelago. Since the Galápagos penguin has been described as one large population due to high levels of gene flow between subpopulations within the archipelago [76], we considered our sampling from two islands as one sample for the species.

### DNA sequencing and genotyping

We performed DNA extractions using either standard phenol-chloroform protocols [78] or Qiagen DNeasy Blood and Tissue Kits (QIAGEN Inc., Valencia, CA, USA). Three types of genetic markers were amplified, including the MHC class II DRβ1 exon 2, mitochondrial (mtDNA) cytochrome c oxidase subunit I (COI), and five microsatellite loci that have been previously tested in both Galápagos and Magellanic penguins [65].

PCR amplification of MHC class II DRβ1 exon 2 was performed using primers Lpen.hum1F2 and Lpen.hum2R originally developed by Kikkawa et al. [79, 80] for *Spheniscus* penguins. We performed PCR amplifications in 25 μl volumes containing approximately 40 ng of genomic DNA, 1X of GoTaq Flexi buffer, 1.5 mM of MgCl_2_, 0.2 mM of each dNTP, 0.5 μM of each primer, and 0.5 units of GoTaq Flexi DNA polymerase (Promega Corp., Madison, WI, USA). The amplification profile included an initial denaturing step for two minutes at 95°C, followed by 27 cycles of one minute at 94°C, one minute at 62°C, and two minutes at 72°C, ending with a final extension for 15 minutes at 72°C [81]. For Galápagos penguin samples, PCR products were purified through standard ethanol precipitation and sent to the University of Chicago DNA sequencing and genotyping facility for direct sequencing. Two homozygote and two heterozygote individuals were then cloned using the pGEM-T Easy Vector System (Promega Corp., Madison, WI, USA) and a minimum of eight independent clones from each individual were selected for sequencing individual alleles (clone sequencing).

To accurately identify alleles of MHC class II DRβ1 exon 2 of each Magellanic penguin individual, DNA sequences from direct sequencing and clone sequencing were compared to each other to help allele confirmation. Likewise, two independent PCRs followed by cloning of PCR products were implemented to ensure true alleles were verifiable in each independent amplification reaction, and to eliminate sequence returns attributable to heteroduplex formation, *Taq* error, or deamination [13, 16, 82]. MHC alleles were confirmed when identical sequences were returned from each independent round of PCR and cloning, as well as when sequences corresponded to an inferred allele from the independent direct sequencing reactions. Therefore, we used a more conservative criterion for MHC allele validation than that proposed by the International Society for Animal Genetics (ISAG) Human, Cattle, and Dog Nomenclature Committees [83, 84, 85].

A section of the mitochondrial COI gene was amplified using primers EM5287 (5’-CACATCAATGAGCTTGCAACTC-3’) and COI-R722 (5’-TAAACTTCAGGGTGACCAAAAAATYA-3’), which have proven to amplify *Spheniscus* mtDNA [75]. We performed PCR amplifications in 25 μl volumes containing approximately 40 ng of genomic DNA, 1X of GoTaq Flexi buffer, 1 mM of MgCl_2_, 0.08 mM of each dNTP, 0.4 μM of each primer, and 0.5 units of GoTaq Flexi DNA polymerase. The amplification profile included an initial denaturing step for three minutes at 94°C, followed by 35 cycles of 30 seconds at 94°C, 30 seconds at 52°C, and 30 seconds at 72°C, ending with a final extension for five minutes at 72°C. Purified PCR products were then sent for sequencing at the University of Chicago.

PCR amplifications of five microsatellite loci (B3-2, G3-6, G2-2, M1-11, and H2-6), originally described by [65], were performed in 25 μl volumes containing approximately 40 ng of genomic DNA, 1X of GoTaq Flexi buffer, 1.5 mM of MgCl_2_, 0.2 mM of each dNTP, 0.4 μM of each primer, and 0.625 units of GoTaq Flexi DNA polymerase. Amplification profiles included an initial denaturing step for two minutes at 95°C, followed by 30 cycles of 45 seconds at 94°C, 45 seconds at the respective annealing temperature, and 45 seconds at 72°C, ending with a final extension for 10 minutes at 72°C. The annealing temperature was 53°C for locus G3-6 and 51°C for all remaining loci (B3-2, G2-2, M1-11 and H2-6). The fluorescently labeled PCR products were then sent for fragment analysis at the University of Chicago.

### Data analysis

We analyzed all sequences and genotypes in Geneious, version R6.1.8 [86]. For MHC sequences, homozygous and heterozygous genotypes were determined through direct sequencing and by comparing direct sequences and clone sequences from each individual. For the MHC data, we calculated the number of alleles, allele frequencies, and Wright’s F_IS_ index [87] using the web version of GENEPOP [88, 89]. In addition, we computed the number of polymorphic sites (S), the average number of nucleotide differences (k), nucleotide diversity (π), and haplotype diversity (h) using DnaSP version 5.10.1 [90]. We assessed observed and expected heterozygosity (*H*_o_ and *H*_e_) and Hardy Weinberg equilibrium in Arlequin version 3.5.2.2 [91]. To test for deviations from mutation-drift equilibrium, we calculated Tajima’s *D* [92] in DnaSP, along with the Ewens-Watterson homozygosity test [93, 94] and Slatkin’s exact test [95] as implemented in Arlequin. To detect historical selection, we calculated nonsynonymous (*d*_N_) and synonymous substitution rates (*d*_S_) for the entire length of the exon, for the putative peptide-binding region (PBR), and for the non-peptide-binding region (non-PBR) using MEGA version 7.0.18 [96] with the Nei-Gojobori method and Jukes-Cantor correction [97]. The putative PBR codons assigned in this study correspond to the 24 peptide-binding region identified for the human class II HLA-DRβ1 molecule [25, 98]. We used the Z-test of selection implemented in MEGA to test if *d*_N_ was significantly larger than *d*_S_.

For the COI data, we used DnaSP to compute the number of haplotypes, haplotype frequencies, the number of polymorphic sites (S), the average number of nucleotide differences (k), nucleotide diversity (π), and haplotype diversity (h). Additionally, we constructed a haplotype network in PopART version 1.7 [99], using the TCS inference method [100], to examine mutational steps leading to the divergence of mtDNA COI haplotypes in the Magellanic and Galápagos penguins. Selection tests included Tajima’s *D* along with *d*_N_/*d*_S_ and Z-test calculations. The Tajima’s *D* and Z-test calculations for the COI data allowed for proper characterization of COI as a neutral marker and thus permitted comparisons between the adaptive MHC marker and the neutral COI marker for the Galápagos and Magellanic penguin sample sets.

For the microsatellite data, we computed number of alleles and allele frequencies, estimated F_IS_ [87] and R_IS_ indices [101], and tested for linkage disequilibrium in GENEPOP. Additionally, we calculated observed and expected heterozygosity and tested for deviations from Hardy-Weinberg equilibrium using Arlequin. For both linkage and Hardy-Weinberg tests, we applied the Bonferroni correction to the alpha value. As with the MHC locus, we performed the Ewens-Watterson homozygosity test and Slatkin’s exact test to assess deviations from mutation-drift equilibrium. The heterozygosity calculations and the Ewens-Watterson test allowed for comparisons between adaptive MHC and neutral microsatellite variation in the Galápagos and Magellanic penguins samples. We implemented a second test of mutation-drift equilibrium using BOTTLENECK, version 1.2.02 [102, 103]. Two models of evolution were assessed in this test: the stepwise mutation model (SMM; [104]) and the two-phase model (TPM; [105]), the latter of which was set to be composed of 90% single step mutations as suggested for microsatellite data [102, 103]. We also performed a Wilcoxon sign-rank test for heterozygosity excess and a mode shift (allele frequency distribution) test [106]. We could only apply BOTTLENECK to the Magellanic penguin samples, which had the required number of polymorphic loci for this analysis. The BOTTLENECK test ensured that the Magellanic penguin samples showed no signs of historical bottlenecks, which would have prevented the use of the Cabo Vírgenes population as an appropriate “control” for comparison to the Galápagos penguin.

## Results

### MHC diversity in Galápagos and Magellanic penguins

The MHC alignment had a final length of 419 bp, with exon 2 spanning bases 93 through 362 (S1 Appendix). There were 19 distinct MHC alleles represented within the 26 Magellanic penguin samples analyzed, whereas only two alleles were detected within the 38 Galápagos penguin samples. The two alleles detected in the Galápagos penguins were distinct from any of the Magellanic penguin alleles (i.e., none of the alleles were shared between the two species). There were 37 polymorphic sites in the Magellanic penguin MHC alleles, whereas the Galápagos penguin MHC revealed only nine polymorphic sites (Table 1). The two alleles from the Galápagos penguin were found at relatively high frequencies (0.605 and 0.395). The observed heterozygosity was significantly higher in the Magellanic penguin (0.923) compared to that of the Galápagos penguin (0.421), although there was no deviation from Hardy-Weinberg equilibrium observed for either sample set (both *P* > 0.5, Table 1). However, the F_IS_ for the Magellanic penguin was -0.012 and for the Galápagos penguin was 0.132, a trend consistent with expectations for large, outbred versus small, possibly inbred populations (Table 2).

**Table 1:** Summary of genetic variation for MHC class II DRβ1 sequences in the Magellanic and Galápagos penguins. Number of samples (N), number of alleles (A), number of polymorphic sites (S), average number of nucleotide differences (k), nucleotide diversity (π), haplotype diversity (h), and observed (*H*_o_) and expected (*H*_e_) heterozygosity are presented. *P*-values (*P*) are reported for tests of deviations from Hardy-Weinberg equilibrium. SD represents standard deviations.

	N	A	S	k	π ± SD	h ± SD	*H*_o_	*H*_e_	*P*
**Magellanic penguin**	26	19	37	13.18	0.031 ± 0.001	0.913 ± 0.024	0.923	0.913	0.980
**Galápagos penguin**	38	2	9	4.36	0.011 ± 0.001	0.484 ± 0.250	0.421	0.484	0.503

**Table 2:** F_IS_ and R_IS_ estimates for Magellanic and Galápagos penguin samples for MHC class II DRβ1 and microsatellite loci. Overall statistics for the microsatellite loci are denoted as Msat All. N/A represent monomorphic microsatellite loci.

	F_IS_		R_IS_	
	Magellanic penguin	Galápagos penguin	Magellanic penguin	Galápagos penguin
**MHC**	-0.012	0.132	N/A	N/A
**Microsatellites**				
**B3-2**	-0.214	N/A	-0.246	N/A
**G3-6**	0.226	-0.121	0.350	-0.121
**G2-2**	-0.160	N/A	-0.022	N/A
**M1-11**	N/A	N/A	N/A	N/A
**H2-6**	-0.136	N/A	-0.136	N/A
**Msat All**	-0.018	-0.121	0.298	-0.121

Analysis of Magellanic penguin MHC sequences revealed higher rates of nonsynonymous (*d*_N_) than synonymous (*d*_S_) substitutions for the entirety of exon 2, and the PBR and non-PBR, resulting in *d*_N_/*d*_S_ ratios significantly larger than one (all *P* < 0.01; Table 3). For the Magellanic penguin samples, the PBR had the highest *d*_N_ value (0.198), although this region also had the highest *d*_S_ value (0.021), resulting in a *d*_N_/*d*_S_ ratio of 9.429. Similar to the Magellanic penguin samples, the Galápagos MHC had significantly higher *d*_N_ than *d*_S_ values along the entirety of exon 2 and for the PBR (both *P* ≤ 0.01). Since there were no synonymous substitutions detected in the Galápagos penguin samples, we could not calculate a *d*_N_/*d*_S_ ratio for this species. Similar to the Magellanic penguin, the Galápagos penguin samples had the highest *d*_N_ value (0.164) within the peptide-binding region. Overall, the Magellanic penguin showed consistently higher *d*_N_ values than the Galápagos penguin across all regions of exon 2. All Z-tests of positive selection were significant for all assessments (*P* ≤ 0.011) except for the Galápagos non-PBR (*P* = 0.163).

**Table 3:** Summary of substitution tests for selection at the MHC class II DRβ1-like gene for the entirety of exon 2, the peptide-binding region (PBR), and the non-peptide-binding region (Non-PBR). Nonsynonymous substitution rate (*d*_N_), synonymous substitution rate (*d*_S_), and *P*-values (*P*) for the Z-test of selection of positive selection are presented.

	Entire Exon 2		PBR		Non-PBR	
	Magellanic penguin	Galápagos penguin	Magellanic penguin	Galápagos penguin	Magellanic penguin	Galápagos penguin
***d*_N_**	0.073	0.045	0.198	0.164	0.034	0.007
***d*_S_**	0.007	0.000	0.021	0.000	0.002	0.000
***d*_N_ / *d*_S_**	10.429	---	9.429	---	17.000	---
***P***	0.000*	0.005*	0.000*	0.011*	0.007*	0.163

* denotes significant probability of *d*_N_ > *d*_S_.

Tajima’s *D* estimates were greater than 2 and significant for both species [Magellanic: *D* = 2.052 (*P* < 0.05); Galápagos *D* = 3.576 (*P* < 0.01)], indicating deviation from mutation-drift equilibrium. The Ewens-Watterson test did not reveal significant differences from neutral expectations, although the difference between the observed and expected homozygosity statistics was found to be greater, but only marginally significant (*P* = 0.091) in the Galápagos penguin (Table 4).

**Table 4:** Summary of Magellanic and Galápagos penguin Ewens-Watterson tests for all MHC class II DRβ1 and microsatellite loci. Ewens-Watterson observed F (*F*_o_), expected F (*F*_e_), and Slatkin’s exact test *P*-values (*P*) are presented. N/A indicates no test was performed because the locus was monomorphic.

	Magellanic penguin			Galápagos penguin		
	*F*_o_	*F*_e_	*P*	*F*_o_	*F*_e_	*P*
**MHC**	0.105	0.102	0.453	0.522	0.799	0.091
**Microsatellites**						
**B3-2**	0.384	0.438	0.407	N/A	N/A	N/A
**G3-6**	0.078	0.128	0.002*	0.791	0.798	0.425
**G2-2**	0.413	0.378	0.904	N/A	N/A	N/A
**M1-11**	N/A	N/A	N/A	N/A	N/A	N/A
**H2-6**	0.767	0.786	0.423	N/A	N/A	N/A

* denotes significant deviation from neutrality.

### Mitochondrial DNA (COI) diversity in Galápagos and Magellanic penguins

**Fig 2:**
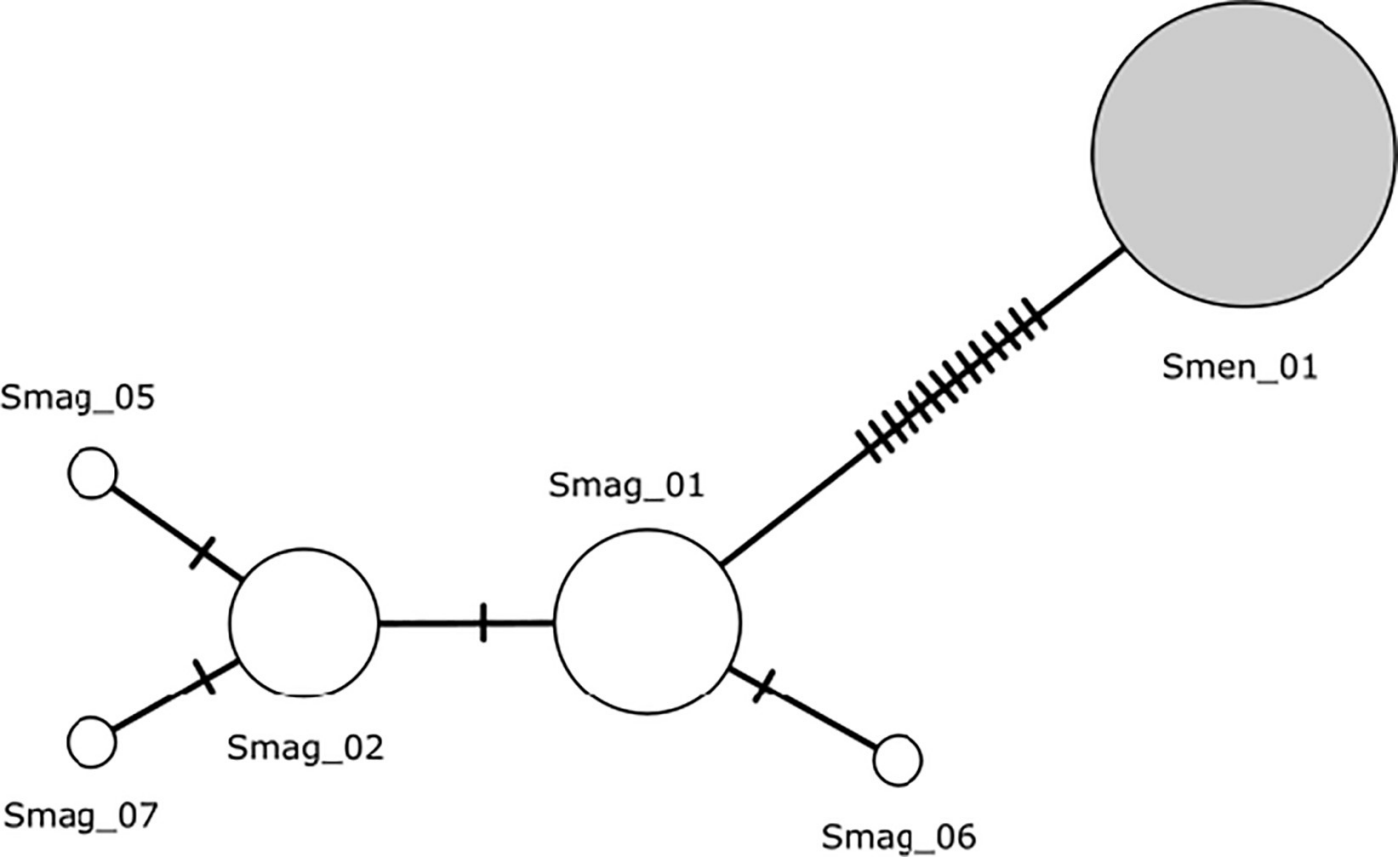
Haplotype network for mitochondrial cytochrome oxidase subunit I (COI) haplotypes detected in Magellanic and Galápagos penguins. The network was constructed under the TCS model in PopART [99]. Dashes along lines represent the number of changes from one haplotype to another. The size of the circle is proportional to the frequency of the haplotype. Magellanic haplotypes (white) are designated as Smag_# and the Galápagos haplotype (gray) is designated as Smen_1.

The COI alignment had a final length of 807 bp, with the first 99 bases covering the tRNA-Tyr gene of the mitochondrial genome (S2 Appendix). There were five distinct haplotypes within the 26 Magellanic penguin samples, and only one haplotype shared among all 38 Galápagos penguin samples analyzed. COI haplotypes detected in the Galápagos and Magellanic penguins were distinct (i.e., there were no shared haplotypes between species). The haplotype network indicated that the Galápagos penguin haplotype had 14 single point mutations separating it from the Magellanic haplotypes, while all the Magellanic haplotypes only differed from each other by one to three changes (Fig 2). Such a large difference between the two species is congruent with the accumulation of mutations after the speciation event that originated both species. In the Magellanic penguin, COI sequences revealed four polymorphic sites, the average number of nucleotide differences (k) was 0.738, the nucleotide diversity (π) was 0.001, and haplotype diversity (h) was 0.609.

Analysis of COI sequences from the Magellanic penguin revealed *d*_S_ was 0.010 whereas *d*_N_ was zero, as there were no nonsynonymous substitutions, resulting in a *d*_N_/*d*_S_ ratio of zero and a nonsignificant Z-test of positive selection (*P* = 1.000). Tajima’s *D* for the Magellanic samples was negative (*D* = -0.790) but not significant (*P* > 0.100), indicative of the locus being in mutation-drift equilibrium. We could not conduct substitution tests or calculate Tajima’s D for the Galápagos penguin COI data due to the monomorphism of the Galápagos samples.

### Microsatellite diversity in Galápagos and Magellanic penguins

There were greater levels of polymorphism uncovered in the 26 Magellanic penguin samples compared to the 38 Galápagos penguin samples analyzed, with only one monomorphic locus (M1-11) detected in the Magellanic penguin and only one polymorphic locus (G3-6) detected in the Galápagos penguin (Table 5). None of the microsatellite loci were in linkage disequilibrium (all *P* > 0.008). The most polymorphic locus, G3-6, had 16 alleles in the Magellanic penguin, but only two alleles (with frequencies 0.882 and 0.118) in the Galápagos penguin (S1 Table). Across all loci, all alleles but one detected in the Galápagos penguin were also found in the Magellanic penguin, with the allele unique to the Galápagos penguin detected at the G3-6 locus. Notably, for the three most polymorphic loci (B3-2, G3-6, and G2-2), the allele fixed or at high frequency in the Galápagos penguin did not correspond to the most frequent allele in the Magellanic penguin (S1 Table). All polymorphic loci were in Hardy-Weinberg equilibrium except for G3-6 for the Magellanic penguin (Table 5).

**Table 5:** Summary of microsatellite data for Magellanic and Galápagos penguins. Number of alleles (A), observed heterozygosity (*H*_o_), expected heterozygosity (*H*_e_), and Hardy-Weinberg equilibrium *P*-values (*P*) for each locus analyzed are presented. N/A indicates no test was performed because the locus was monomorphic.

	Magellanic penguin				Galápagos penguin			
	A	*H*_o_	*H*_e_	*P*	A	*H*_o_	*H*_e_	*P*
								
**B3-2**	5	0.760	0.629	0.398	1	0.000	0.000	N/A
**G3-6**	16	0.731	0.940	0.005*	2	0.237	0.212	1.000
**G2-2**	6	0.692	0.599	0.438	1	0.000	0.000	N/A
**M1-11**	1	0.000	0.000	N/A	1	0.000	0.000	N/A
**H2-6**	2	0.269	0.238	1.000	1	0.000	0.000	N/A
**Mean**	6	0.490	0.481	N/A	1.2	0.047	0.042	N/A

* denotes significant deviation from Hardy-Weinberg equilibrium (*P* < 0.01).

The Ewens-Watterson test did not reveal differences from neutral expectations except at the G3-6 locus in the Magellanic penguin, where the observed homozygosity statistic (0.078) was significantly lower than the expected value (0.128), suggestive of an allele distribution that is more even than anticipated under neutrality (Table 4). In the BOTTLENECK analysis, the Wilcoxon sign-rank tests under both the stepwise mutation model (SMM) and two-phase model (TPM) were not indicative of heterozygosity excess in the Magellanic penguin (SMM and TPM *P*-values = 0.906). Instead, the Magellanic penguin samples displayed a normal L-shaped distribution as expected under mutation-drift equilibrium. We could not perform a BOTTLENECK analysis on the Galápagos penguin data given that only one microsatellite locus was polymorphic.

## Discussion

We compared the genetic diversity present at neutral and adaptive loci in the demographically bottlenecked Galápagos penguin with that of its abundant congener, the Magellanic penguin. It is clear that Galápagos penguins are depauperate in genetic diversity compared to Magellanic penguins at all loci examined, consistent with their low abundance, restricted geographic distribution, and the effects of undergoing serial demographic bottleneck events throughout their evolutionary history. The low diversity evidenced at the microsatellite and MHC loci is also consistent with the findings of prior Galápagos penguin studies [11, 65, 76]. In the Galápagos penguin, there were considerably fewer alleles across all loci examined and lower levels of heterozygosity at microsatellites and MHC than the Magellanic penguin, both of which are consistent with bottleneck theory expectations indicative of genetic drift acting during drastic demographic declines [3, 4]. These observations support the hypothesis that the restricted distribution of the Galápagos penguin associated with its history of serial demographic bottlenecks is, in part, the cause of their decreased genetic diversity. The high levels of genetic variation and the absence of bottleneck signatures from the BOTTLENECK analysis of the Magellanic penguin indicates that this population has not experienced significant demographic declines, further validating the use of this population as a non-bottlenecked “control” for comparison.

Although demographic bottlenecks are known to decrease genetic diversity, we cannot rule out the possibility that the restricted distribution and relatively low abundance of the Galápagos penguin over evolutionary time has contributed to the low levels of genetic diversity documented in this species. While genetic drift may have led to a decrease in heterozygosity and the retention of fewer alleles across multiple loci, our results also show both historical and recent signatures of selection at the MHC class II DRβ1 locus. We detected historical selection (i.e., selection over an evolutionary time scale) at MHC loci through *d*_N_/*d*_S_ ratios greater than one and a large, positive Tajima’s *D* statistic. As is commonly found when evaluating MHC loci, *d*_N_ was significantly larger than *d*_S_ for both the Galápagos and Magellanic penguins along the entirety of exon 2, and more specifically within the PBR, evidencing historical positive selection acting at this gene. It is interesting to note that, while the rate of synonymous substitutions was low in the Magellanic penguin samples (exon 2 *d*_S_ = 0.007), there were zero synonymous substitutions present in the Galápagos penguin, indicating that all polymorphic sites in the Galápagos MHC sequences resulted from substitutions that produced amino acid changes. Such absence of synonymous substitutions was also found in the Galápagos penguin samples tested by Bollmer et al. [11]. The other test for historical selection, the Tajima’s *D* statistic, specifically identifies potential signals of balancing selection. Both Magellanic and Galápagos penguins showed evidence of deviations from mutation-drift equilibrium, revealed by large, positive *D* statistics and significant *P*-values. Tajima’s *D* is influenced by selection and demographic events [107], thus significant deviations from mutation-drift equilibrium yielding a positive *D* value could be interpreted as either evidence of balancing selection or past bottleneck events [108]. The Tajima’s test results for the Magellanic penguin are best explained by past balancing selection maintaining diversity at the MHC, since there were no signs of genetic bottlenecks in the BOTTLENECK analysis. The larger *D* statistic of Galápagos penguins (*D* = 3.576) compared to that of the Magellanic penguin (*D* = 2.052) suggests that either balancing selection was more intense in the Galápagos penguin or, alternatively, bottleneck effects and balancing selection both contributed to increasing the Tajima’s *D* statistic.

Recent balancing selection (i.e., contemporary signals of selection) can be detected either through an excess of heterozygote genotypes compared to Hardy-Weinberg expectations or by finding a significantly lower observed homozygosity than expected under mutation-drift equilibrium in the Ewens-Watterson test. Our results from these analyses did not reveal recent balancing selection acting on the MHC locus in either the Magellanic or the Galápagos penguin. The Hardy-Weinberg *P*-values were not significant for either species, showing no heterozygote excess. However, conformity with Hardy-Weinberg expectations may be due to the potential inability to detect selection within a single generation, or to selection events operating in some generations but not others [109]. Although *F*_o_ and *F*_*e*_ calculated by the Ewens-Watterson test were not significantly different in the Magellanic penguin (*P* = 0.453), for the Galápagos penguin there was a marginally significant result (*P* = 0.091). A lower *F*_o_ (0.522) than *F*_*e*_ (0.799) in the Galápagos penguins indicates that allele frequencies are more evenly distributed than expected under mutation-drift equilibrium and suggests possible balancing selection. The lack of significance in the Ewens-Watterson test may be because the test relies on allele frequencies, not divergence information, and may thus underestimate current signatures of balancing selection [109].

Indirect evidence for recent balancing selection (e.g., during bottleneck events) may be based on patterns of MHC polymorphisms compared to neutral markers, such as finding greater numbers of alleles, more even allele frequencies, and higher heterozygosity levels at the MHC locus than at neutral loci such as microsatellites [39, 110]. Hardy-Weinberg and Ewens-Watterson tests did not show evidence of recent balancing selection. However, higher levels of heterozygosity in the MHC compared to neutral loci suggest that MHC alleles are maintained through balancing selection. For example, the MHC locus in the Galápagos penguin samples had an *H*_o_ of 0.421, while the average *H*_o_ across all microsatellite loci was an order of magnitude lower (0.047). In fact, MHC heterozygosity in the Galápagos penguin was still twice that detected for the only polymorphic microsatellite locus (G3-6; *H*_o_ = 0.237), even though both loci harbored the same number of alleles (two). The detection of higher levels of heterozygosity at the MHC locus compared to the typically highly polymorphic and neutral microsatellite loci suggests that selection has played an important role in maintaining genetic diversity at the MHC, even when the population has undergone serial demographic bottlenecks. However, caution must be taken since these expectations are based on the assumption that both adaptive and neutral loci had similar numbers of alleles and allele frequencies prior to the bottleneck events [4].

In addition to higher heterozygosity, the number of alleles and evenness of allele frequencies was greater in both penguin species at the MHC locus compared to these measures at the neutral loci analyzed. In the Galápagos penguin, there were approximately two times as many MHC alleles compared to microsatellite alleles and COI haplotypes. Likewise, there were approximately four times as many MHC alleles compared to the number of alleles at neutral markers observed in the Magellanic penguin. As indicated above, only one locus was polymorphic in the Galápagos penguin, which had two alleles observed at disparate frequencies (0.882 and 0.118). In contrast, the two MHC alleles had frequencies of 0.605 and 0.395, consistent with the idea that balancing selection may have played a role in maintaining their relatively high and even frequencies, thus counteracting the dominant effects of genetic drift during bottleneck events.

We found evidence of both historical (*d*_N_/*d*_S_ ratio and Tajima’s *D*) and recent selection (heterozygosity, number of alleles, and evenness of allele frequencies) acting at the MHC locus. Several studies on natural populations have found evidence of historical but not recent balancing selection at MHC loci (e.g., [25, 26, 109, 111]). Furthermore, most studies of MHC variation in bottlenecked populations conclude that genetic drift generally outweighs recent balancing selection during demographic bottlenecks (e.g., [25, 26, 109, 111, 112, 113, 114]). Our results suggest that the commonly held view of the dominant role of genetic drift during bottleneck events may not be a general rule. Contrasting patterns of polymorphisms between adaptive (MHC) and neutral (microsatellites and mitochondrial) loci suggest that balancing selection at the MHC class II DRβ1 locus can ameliorate the effects of genetic drift, at least in the Galápagos penguin. The active role of selection during bottleneck events has been previously demonstrated in experimental populations of *Drosophila melanogaster* [45, 46]. A few other studies have found recent signatures of selection at MHC loci in natural populations despite the presence of bottleneck events (e.g., [36, 37, 38, 39, 40, 41]). Our study adds, therefore, to the body of literature suggesting that selection can potentially mitigate the effects of genetic drift during demographic bottlenecks.

### Conservation implications

We suggest that the restricted distribution and relatively low abundance of Galápagos penguins throughout their evolutionary history, in addition to the effects of demographic bottlenecks associated with recurring ENSO events [49, 53, 54], have significantly reduced the overall genetic diversity of the species. Although previous studies provided evidence that Galápagos penguins have reduced genetic diversity at different loci [11, 65, 76], the independent analyses of samples each with different types of genetic markers limited potential inferences about the role of genetic drift and selection in maintaining or reducing genetic diversity. Furthermore, inferences about the role of demographic history on genetic diversity were also limited by the lack of a proper non-bottlenecked population “control” that accounts for the Galápagos penguin’s restricted geographic distribution.

Our study shows that the Galápagos penguin represents another emblematic case of the potential effect of environmental changes driving demographic declines, which have direct consequences on the genetic diversity of species. The concurrent analyses of both neutral and adaptive markers, as well as markers with distinct modes of inheritance, consistently showed that the Galápagos penguin has significantly reduced genetic diversity at all loci analyzed, as compared with a reference population of Magellanic penguins with no documented history of bottlenecks. Most notably, however, the results indicate that balancing selection may have played an essential role in maintaining diversity at the MHC class II DRβ1-like gene in the Galápagos penguin. Although loss of MHC variation may place endangered species at higher risk of susceptibility to novel pathogens [115], many bottlenecked populations have persisted for long periods and not shown decreased population viability (e.g., [25, 34, 112, 114, 116, 117]). Survival of populations depends in part on pathogen load and other characteristics of the species [26]. Although the Galápagos penguin is an opportunistic breeder that recovers its population size rather quickly after bottleneck events [49, 50], the survival of the species will in part depend on whether the diversity at the MHC is sufficient to maintain the population in the face of potential diseases and parasites. While the alleles present may be adequate to counter the pathogens and parasites currently found in the Galápagos Islands, the species may easily become vulnerable to novel diseases. More troubling is the presence of *Plasmodium* parasites, known to be a vector for avian malaria, which have been found in blood samples of Galápagos penguins [118].

Although the Galápagos penguin may be adapted to cyclic changes in population size over evolutionary time, it seems clear that anthropogenic events leading to increases in pathogen exposure and increased frequency of El Niño events due to global climate change, may put this species at further risk of extinction, particularly in light of their overall low levels of genetic diversity. Therefore, continued management and protection of this species is essential for their long-term conservation, in addition to continued assessment of pathogen exposure.

## Supporting information

S1 Appendix Alignment of MHC class II DRβ1 alleles from Magellanic and Galápagos samples analyzed. (DOCX)

S2 Appendix Alignment of mitochondrial COI haplotypes from Magellanic and Galápagos samples analyzed. (DOCX)

S1 Table Allele frequencies observed at the five microsatellite loci (B3-2, G3-6, G2-2, M1-11, and H2-6) assessed for the Magellanic and Galápagos penguins. (DOCX)
