# Supplementary material for: The role of demographic history and selection in shaping genetic diversity of the Galápagos penguin (Spheniscus mendiculus)
Source: PLoS One. 2020 Jan 7;15(1):e0226439. doi: 10.1371/journal.pone.0226439 (PMC6946592; doi:10.1371/journal.pone.0226439)
Supplement: S1 Appendix — (DOCX) [file pone.0226439.s001.docx]

**S1 Appendix**

**Alignment of MHC class II DRβ1 alleles from Magellanic and Galápagos samples analyzed.** Magellanic alleles are designated as Smag_# and Galápagos alleles are designated as Smen_#. The starting and ending positions, bases 93 and 362, of exon 2 are marked with > and < signs above them, respectively. Putative peptide-binding codons (based on [85]) are marked with asterisks (*) above the corresponding base positions.

**> *** *** *****

Smag_01 GCCATGGGGGGGGCTGGGAGGAGAGCGGCAACGGGGAGGGGGCTCCACGGTGCCTCCCTGCCCACCCACGACTTGCCTCCCTGCGCAAACAGGGTATTTCCAGGAGATGCTTAAGGCCGA

Smag_02 GCCATGGGGGGGGCTGGGAGGAGAGCGGCAACGGGGAGGGGGCTCCACGGTGCCTCCCTGCCCACCCACGACTTGCCTCCCTGCGCAAACAGGGTATTTCCAGGAGATGGGTAAGGCCGA

Smag_03 GCCATGGGGGGGGCTGGGAGGAGAGCGGCAACGGGGAGGGGGCTCCACGGTGCCTCCCTGCCCACCCACGACTTGCCTCCCTGCGCAAACAGGGTATTTCCAGGAGATGGGTAAGTTCGA

Smag_04 GCCATGGGGGGGGCTGGGAGGAGAGCGGCAACGGGGAGGGGGCTCCACGGTGCCTCCCTGCCCACCCACGACTTGCCTCCCTGCGCAAACAGGGTATTTCCAGGAGATGGGTAAGGCCGA

Smag_05 GCCATGGGGGGGGCTGGGAGGAGAGCGGCAACGGGGAGGGGGCTCCACGGTGCCTCCCTGCCCACCCACGACTTGCCTCCCTGCGCAAACAGGGTATTTCCAGGAGATGGGTAAGTTCGA

Smag_06 GCCATGGGGGGGGCTGGGAGGAGAGCGGCAACGGGGAGGGGGCTCCACGGTGCCTCCCTGCCCACCCACGACTTGCCTCCCTGCGCAAACAGGGTATTTCCAGGAGATGCTTAAGGCCGA

Smag_07 GCCATGGGGGGGGCTGGGAGGAGAGCGGCAACGGGGAGGGGGCTCCACGGTGCCTCCCTGCCCACCCACGACTTGCCTCCCTGCGCAAACAGGGTATTTCCAGGAGATGCTTAAGGCCGA

Smag_08 GCCATGGGGGGGGCTGGGAGGAGAGCGGCAACGGGGAGGGGGCTCCACGGTGCCTCCCTGCCCACCCACGACTTGCCTCCCTGCGCAAACAGGGTATTTCCAGGAGATGGGTAAGGCCGA

Smag_09 GCCATGGGGGGGGCTGGGAGGAGAGCGGCAACGGGGAGGGGGCTCCACGGTGCCTCCCTGCCCACCCACGACTTGCCTCCCTGCGCAAACAGGGTATTTCCAGGAGATGCTTAAGTTCGA

Smag_11 GCCATGGGGGGGGCTGGGAGGAGAGCGGCAACGGGGAGGGGGCTCCACGGTGCCTCCCTGCCCACCCACGACTTGCCTCCCTGCGCAAACAGGGTATTTCCAGGAGATGGGTAAGTTCGA

Smag_12 GCCATGGGGGGGGCTGGGAGGAGAGCGGCAACGGGGAGGGGGCTCCACGGTGCCTCCCTGCCCACCCACGACTTGCCTCCCTGCGCAAACAGGGTATTTCCAGGAGATGGGTAAGTTCGA

Smag_15 GCCATGGGGGGGGCTGGGAGGAGAGCGGCAACGGGGAGGGGGCTCCACGGTGCCTCCCTGCCCACCCACGACTTGCCTCCCTGCGCAAACAGGGTATTTCCAGGAGATGGGTAAGTCCGA

Smag_18 GCCATGGGGGGGGCTGGGAGGAGAGCGGCAACGGGGAGGGGGCTCCACGGTGCCTCCCTGCCCACCCACGACTTGCCTCCCTGCGCAAACAGGGTATTTCCAGGAGATGGGTAAGGCCGA

Smag_20 GCCATGGGGGGGGCTGGGAGGAGAGCGGCAACGGGGAGGGGGCTCCACGGTGCCTCCCTGCCCACCCACGACTTGCCTCCCTGCGCAAACAGGGTATTTCCAGGAGATGCATAAGTCCAA

Smag_23 GCCATGGGGGGGGCTGGGAGGAGAGCGGCAACGGGGAGGGGGCTCCACGGTGCCTCCCTGCCCACCCACGACTTGCCTCCCTGCGCAAACAGGGTATTTCCAGGAGATGCATAAGGCCGA

Smag_24 GCCATGGGGGGGGCTGGGAGGAGAGCGGCAACGGGGAGGGGGCTCCACGGTGCCTCCCTGCCCACCCACGACTTGCCTCCCTGCGCAAACAGGGTATTTCCAGGAGATGGGTAAGGCCGA

Smag_27 GCCATGGGGGGGGCTGGGAGGAGAGCGGCAACGGGGAGGGGGCTCCACGGTGCCTCCCTGCCCACCCACGACTTGCCTCCCTGCGCAAACAGGGTATTTCCAGGAGATGCATAAGTCCAA

Smag_37 GCCATGGGGGGGGCTGGGAGGAGAGCGGCAACGGGGAGGGGGCTCCACGGTGCCTCCCTGCCCACCCACGACTTGCCTCCCTGCGCAAACAGGGTATTTCCAGGAGATGGGTAAGGCCGA

Smag_39 GCCATGGGGGGGGCTGGGAGGAGAGCGGCAACGGGGAGGGGGCTCCACGGTGCCTCCCTGCCCACCCACGACTTGCCTCCCTGCGCAAACAGGGTATTTCCAGGAGATGCTTAAGGCCGA

Smen_01 GCCATGGGGGGGGCTGGGAGGAGAGCGGCAACGGGGAGGGGGCTCCACGGTGCCTCCCTGCCCACCCACGACTTGCCTCCCTGCGCAAACAGGGTATTTCCAGAGGATGCTTAAGTTCGA

Smen_02 GCCATGGGGGGGGCTGGGAGGAGAGCGGCAACGGGGAGGGGGCTCCACGGTGCCTCCCTGCCCACCCACGACTTGCCTCCCTGCGCAAACAGGGTATTTCCAGGAGATGGTTAAGGCCGA

*** *** *** ****** ***

Smag_01 GTGTCATTTCCTCAACGGCACCGAGCGGGTGAGGTTTGTGGTGAGGGACATCTACAACCGGCAGCAGGACGTGCACTTCGACAGCGACGTGGGGCAGTTTGTGGCCGACACCCCCCTGGG

Smag_02 GTGTCATTTCCTCAACGGCACCGAGCGGGTGAGGTTTGTGGAGAGGTACCTCTACAACCGGCAGCAGTACGTGCACTTCGACAGCGACGTGGGGTACTATGTGGCCGACACCCCCCTGGG

Smag_03 GTGTCATTTCCTCAACGGCACCGAGCGGGTGAGGTTTGTGGACAGGTACATCTACAACCGGCAGCAGTACGTGCACTTCGACAGCGACGTGGGGCAGTTTGTGGCCGACACCCCCCTGGG

Smag_04 GTGTCATTTCCTCAACGGCACCGAGCGGGTGAGGTTTGTGGACAGGTACATCTACAACCGGCAGGAGTACGTGCACTTCGACAGCGACGTGGGGCAGTTTGTGGCCGACACCCCCCTGGG

Smag_05 GTGTCATTTCCTCAACGGCACCGAGCGGGTGAGGTTTGTGGACAGGTACATCTACAACCGGCAGCAGTACGTGCACTTCGACAGCGACGTGGGGTACTATGTGGCCGACACCCCCCTGGG

Smag_06 GTGTCATTTCCTCAACGGCACCGAGCGGGTGAGGTATGTGGTGAGGGACATCTACAACCGGCAGCAGAACGTGCACTTCGACAGCGACGTGGGGCAGTTTGTGGCCGACACCCCCCTGGG

Smag_07 GTGTCATTTCCTCAACGGCACCGAGCGGGTGAGGTTTGTGGTGAGGGACATCTACAACCGGCAGCAGGACGTGCACTTCGACAGCGACGTGGGGTACTATGTGGCCGACACCCCCCTGGG

Smag_08 GTGTCATTTCCTCAACGGCACCGAGCGGGTGAGGTTTGTGGACAGGTACATCTACAACCGGCAGGAGTACGTGCACTTCGACAGCGACGTGGGGCAGTTTGTGGCCGACACCCCCCTGGG

Smag_09 GTGTCATTTCCTCAACGGCACCGAGCGGGTGAGGTATGTGGTGAGGGACATCTACAACCGGCAGCAGAACGTGCACTTCGACAGCGACGTGGGGCAGTTTGTGGCCGACACCCCCCTGGG

Smag_11 GTGTCATTTCCTCAACGGCACCGAGCGGGTGAGGTTTGTGGACAGGTACATCTACAACCGGCAGCAGTACGTGCACTTCGACAGCGACGTGGGGTACTATGTGGCCGACACCCCCCTGGG

Smag_12 GTGTCATTTCCTCAACGGCACCGAGCGGGTGAGGTTTGTGGACAGGTACATCTACAACCGGCAGCAGTACGTGCACTTCGACAGCGACGTGGGGCAGTTTGTGGCCGACACCCCCCTGGG

Smag_15 GTGTCATTTCCTCAACGGCACCGAGCGGGTGAGGTTTGTGGACAGGTACATCTACAACCGGCAGCAGTACGTGCACTTCGACAGCGACGTGGGGTACTATGTGGCCGACACCCCCCTGGG

Smag_18 GTGTCATTTCCTCAACGGCACCGAGCGGGTGAGGTATGTGGAGAGGTACATCTACAACCGGCAGGAGTACGTGCACTTCGACAGCGACGTGGGGTACTATGTGGCCGACACCCCCCTGGG

Smag_20 GTGTCATTTCCTCAACGGCACCGAGCGGGTGAGGTATGTGGAGAGGTACATCTACAACCGGCAGCAGGACGTGCACTTCGACAGCGACGTGGGGTACTATGTGGCCGACACCCCCCTGGG

Smag_23 GTGTCATTTCCTCAACGGCACCGAGCGGGTGAGGCTTGTGGAGAGGTACATCTACAACCGGCAGCAGTACGTGCACTTCGACAGCGACGTGGGGCAGTTTGTGGCCGACACCCCCCTGGG

Smag_24 GTGTCATTTCCTCAACGGCACCGAGCGGGTGAGGTTTGTGGACAGGTACATCTACAACCGGCAGGAGTACGTGCACTTCGACAGCGACGTGGGGCAGTTTGTGGCCGACACCCCCCTGGG

Smag_27 GTGTCATTTCCTCAACGGCACCGAGCGGGTGAGGTATGTGGAGAGGTACATCTACAACCGGCAGCAGGACGTGCACTTCGACAGCGACGTGGGGTACTATGTGGCCGACACCCCCCTGGG

Smag_37 GTGTCATTTCCTCAACGGCACCGAGCGGGTGAGGTTTGTGGACAGGTACATCTACAACCGGCAGGAGTACGTGCACTTCGACAGCGACGTGGGGCAGTTTGTGGCCGACACCCCCCTGGG

Smag_39 GTGTCATTTCCTCAACGGCACCGAGCGGGTGAGGTTTGTGGACAGGTACATCTACAACCGGCAGGAGTACGTGCACTTCGACAGCGACGTGGGGCAGTTTGTGGCCGACACCCCCCTGGG

Smen_01 GTGTCATTTCCTCAACGGCACCGAGCGGGTGAGGTATGTGGAGAGGGACATCTACAACCGGCAGCAGGACGTGCACTTCGACAGCGACGTGGGGTACTATGTGGCCGACACCCCCCTGGG

Smen_02 GTGTCATTTCCTCAACGGCACCGAGCGGGTGAGGTTTGTGGTGAGGGACATCTACAACCGGCAGCAGGACGTGCACTTCGACAGCGACGTGGGGTACTATGTGGCCGACACCCCCCTGGG

*** ****** *** *** ****** *** *** ****** ****** ******

Smag_01 TGAGCCTGATGCCAAGTACTGGAACAGCCAGACGGACTTCCTGGAGCAGAGCCGGGCTGCGGTGGACACGATCTGCCGACACAACTACGGGGTGGTGACCCCTTTCACTGTGGAGAGGAG

Smag_02 TGAGCCTTCTGCCAAGTACTGGAACAGCCAGACGGACTTACTGGAGCAGAAACGGGCTGAGGTGGACACGTACTGCCGACACAACTACGGGGTGGTGACCCCTTTCACTGTGGAGAGGAG

Smag_03 TGAGCTTATTGCCAAGTACTTGAACAGCCAGACGGACTTACTGGAGCAGACACGGGCTGCGGTGGACACGTACTGCCGACACAACTACGGGGTGGTGACCCCTTTCACTGTGGAGAGGAG

Smag_04 TGAGCCTTCTGCCAAGTACTGGAACAGCCAGACGGACATACTGGAGGATGAACGGGCTGCGGTGGACACGTACTGCCGACACAACTACGGGGTGTTTACCCCTTTCACTGTGGAGAGGAG

Smag_05 TGAGCCTTCTGCCAAGTACTTGAACAGCCAGACGGACTTCCTGGAGCAGAAACGGGCTGCGGTGGACAGGTACTGCCGACACAACTACGGGGTGGGGACCCCTTTCACTGTGGAGAGGAG

Smag_06 TGAGCTTATTGCCAAGTACTGGAACAGCCAGACGGACTTACTGGAGCAGAGACGGGCTGAGGTGGACACGGTCTGCCGACACAACTACGGGGTGGTGACCCCTTTCACTGTGGAGAGGAG

Smag_07 TGAGCCTGATGCCAAGTACTGGAACAGCCAGACGGACTTCCTGGAGCAGAGCCGGGCTGCGGTGGACACGATCTGCCGACACAACTACGGGGTGTTTACCCCTTTCACTGTGGAGAGGAG

Smag_08 TGAGCTTATTGCCAAGTACTTGAACAGCCAGACGGACTTACTGGAGCAGAGACGGGCTGAGGTGGACAGGTACTGCCGACACAACTACGGGGTGGTGACCCCTTTCACTGTGGAGAGGAG

Smag_09 TGAGCTTATTGCCAAGTACTGGAACAGCCAGACGGACTTCCTGGAGCAGAAACGGGCTGAGGTGGACACGGTCTGCCGACACAACTACGGGGTGGTGACCCCTTTCACTGTGGAGAGGAG

Smag_11 TGAGCCTTCTGCCAAGTACTTGAACAGCCAGACGGACTTACTGGAGCAGAGACGGGCTGAGGTGGACACGTACTGCCGACACAACTACGGGGTGGTGACCCCTTTCACTGTGGAGAGGAG

Smag_12 TGAGCTTATTGCCAAGTACTTGAACAGCCAGACGGACTTACTGGAGCAGAGACGGGCTGCGGTGGACACGTACTGCCGACACAACTACGGGGTGGTGACCCCTTTCACTGTGGAGAGGAG

Smag_15 TGAGCCTTCTGCCAAGTACTTGAACAGCCAGACGGACTTACTGGAGCAGAGACGGGCTGAGGTGGACACGTACTGCCGACACAACTACGGGGTGGTGACCCCTTTCACTGTGGAGAGGAG

Smag_18 TGAGCCTGATGCCAAGTACTGGAACAGCCAGACGGACATACTGGAGCGGAAACGGGCTGCGGTGGACACGTACTGCCGACACAACTACGGGGTGGGGACCCCTTTCACTGTGGAGAGGAG

Smag_20 TGAGCCTGATGCCAAGTACTGGAACAGCCAGACGGACATACTGGAGCGGAAACAGGCTGCGGTGGACAGGTACTGCCGACACAACTACGGGGTGTTTACCCCTTTCACTGTGGAGAGGAG

Smag_23 TGAGCTTATTGCCAAGTACTGGAACAGCCAGACGGACTTACTGGAGCAGAGACGGGCTGCGGTGGACACGTACTGCCGACACAACTACGGGGTGGTGACCCCTTTCACTGTGGAGAGGAG

Smag_24 TGAGCTTATTGCCAAGTACTTGAACAGCCAGACGGACATACTGGAGGATGAACGGGCTGAGGTGGACAGGTACTGCCGACACAACTACGGGGTGGTGACCCCTTTCACTGTGGAGAGGAG

Smag_27 TGAGCCTGATGCCAAGTACTGGAACAGCCAGACGGACTTCCTGGAGCAGAAACGGGCTGCGGTGGACAGGTACTGCCGACACAACTACGGGGTGTTTACCCCTTTCACTGTGGAGAGGAG

Smag_37 TGAGCTTATTGCCAAGTACTGGAACAGCCAGACGGACATACTGGAGGATGAACGGGCTGCGGTGGACACGTACTGCCGACACAACTACGGGGTGGTGACCCCTTTCACTGTGGAGAGGAG

Smag_39 TGAGCTTATTGCCAAGTACTGGAACAGCCAGACGGACATACTGGAGGATGAACGGGCTGAGGTGGACAGGTACTGCCGACACAACTACGGGGTGTTTACCCCTTTCACTGTGGAGAGGAG

Smen_01 TGAGCCTGATGCCAAGTACTGGAACAGCCAGACGGACATACTGGAGCAGAGACGGGCTGCGGTGGACACGTACTGCCGACACAACTACGGGGTGGTGACCCCTTTCACTGTGGAGAGGAG

Smen_02 TGAGCCTGATGCCAAGTACTGGAACAGCCAGACGGACATACTGGAGCAGAGACGGGCTGAGGTGGACACGTACTGCCGACACAACTACGGGGTGGGGACCCCTTTCACTGTGGAGAGGAG

<

Smag_01 AGGTGAGTGCGTGGCAGAACATCTCCCCGGGGGACGGGCGCAAGCCAAGCCCCGGGGCT

Smag_02 AGGTGAGTGCGTGGCAGAACATCTCCCCGGGGGACGGGCGCAAGCCAAGCCCCGGGGCT

Smag_03 AGGTGAGTGCGTGGCAGAACATCTCCCCGGGGGACGGGCGCAAGCCAAGCCCCGGGGCT

Smag_04 AGGTGAGTGCGTGGCAGAACATCTCCCCGGGGGACGGGCGCAAGCCAAGCCCCGGGGCT

Smag_05 AGGTGAGTGCGTGGCAGAACATCTCCCCGGGGGACGGGCGCAAGCCAAGCCCCGGGGCT

Smag_06 AGGTGAGTGCGTGGCAGAACATCTCCCCGGGGGACGGGCGCAAGCCAAGCCCCGGGGCT

Smag_07 AGGTGAGTGCGTGGCAGAACATCTCCCCGGGGGACGGGCGCAAGCCAAGCCCCGGGGCT

Smag_08 AGGTGAGTGCGTGGCAGAACATCTCCCTGGGGGACGGGCGCAAGCCAAGCCCCGGGGCT

Smag_09 AGGTGAGTGCGTGGCAGAACATCTCCCCGGGGGACGGGCGCAAGCCAAGCCCCGGGGCT

Smag_11 AGGTGAGTGCGTGGCAGAACATCTCCCCGGGGGACGGGCGCAAGCCAAGCCCCGGGGCT

Smag_12 AGGTGAGTGCGTGGCAGAACATCTCCCCGGGGGACGGGCGCAAGCCAAGCCCCGGGGCT

Smag_15 AGGTGAGTGCGTGGCAGAACATCTCCCCGGGGGACGGGCGCAAGCCAAGCCCCGGGGCT

Smag_18 AGGTGAGTGCGTGGCAGAACATCTCCCCGGGGGACGGGCGCAAGCCAAGCCCCGGGGCT

Smag_20 AGGTGAGTGCGTGGCAGAACATCTCCCCGGGGGACGGGCGCAAGCCAAGCCCCGGGGCT

Smag_23 AGGTGAGTGCGTGGCAGAACATCTCCCCGGGGGACGGGCGCAAGCCAAGCCCCGGGGCT

Smag_24 AGGTGAGTGCGTGGCAGAACATCTCCCCGGGGGACGGGCGCAAGCCAAGCCCCGGGGCT

Smag_27 AGGTGAGTGCGTGGCAGAACATCTCCCCGGGGGACGGGCGCAAGCCAAGCCCCGGGGCT

Smag_37 AGGTGAGTGCGTGGCAGAACATCTCCCCGGGGGACGGGCGCAAGCCAAGCCCCGGGGCT

Smag_39 AGGTGAGTGCGTGGCAGAACATCTCCCCGGGGGACGGGCGCAAGCCAAGCCCCGGGGCT

Smen_01 AGGTGAGTGCGTGGCAGAACATCTCCCCGGGGGACGGGCGCAAGCCAAGCCCCGGGGCT

Smen_02 AGGTGAGTGCGTGGCAGAACATCTCCCCGGGGGACGGGCGCAAGCCAAGCCCCGGGGCT
