# Supplementary material for: The role of demographic history and selection in shaping genetic diversity of the Galápagos penguin (Spheniscus mendiculus)
Source: PLoS One. 2020 Jan 7;15(1):e0226439. doi: 10.1371/journal.pone.0226439 (PMC6946592; doi:10.1371/journal.pone.0226439)
Supplement: S2 Appendix — (DOCX) [file pone.0226439.s002.docx]

**S2 Appendix**

**Alignment of mitochondrial COI haplotypes from Magellanic and Galápagos samples analyzed.** Magellanic haplotypes are designated as Smag_# and the Galápagos haplotype is designated as Smen_#. The starting and ending positions of the COI region, bases 100 and 807, are marked with > and < signs above them, respectively.

**>**

Smag_01 AACATGAATTTCACTACAGAACCGATAAGAAGAGGAATCAAACCTCTGTGAAAAGGACTACAGCCTAACGCCTTAACATTCGGCCATCTTACCTTACCTGTGACCTTCATCAACCGATGA

Smag_02 AACATGAATTTCACTACAGAACCGATAAGAAGAGGAATCAAACCTCTGTGAAAAGGACTACAGCCTAACGCCTTAACATTCGGCCATCTTACCTTACCTGTGACCTTCATCAACCGATGA

Smag_05 AACATGAATTTCACTACAGAACCGATAAGAAGAGGAATCAAACCTCTGTGAAAAGGACTACAGCCTAACGCCTTAACATTCGGCCATCTTACCTTACCTGTGACCTTCATCAACCGATGA

Smag_06 AACATGAATTTCACTACAGAACCGATAAGAAGAGGAATCAAACCTCTGTGAAAAGGACTACAGCCTAACGCCTTAACATTCGGCCATCTTACCTTACCTGTGACCTTCATCAACCGATGA

Smag_07 AACATGAATTTCACTACAGAACCGATAAGAAGAGGAATCAAACCTCTGTGAAAAGGACTACAGCCTAACGCCTTAACATTCGGCCATCTTACCTTACCTGTGACCTTCATCAACCGATGA

Smen_01 AACATGAATTTCACTACAGAACCGATAAGAAGAGGAATCAAACCTCTGTGAAAAGGACTACAGCCTAACGCCTTAACATTCAGCCATCTTACCTTACCTGTGACCTTCATCAACCGATGA

Smag_01 TTATTCTCAACCAACCACAAAGATATCGGCACCCTTTACCTAATCTTCGGCGCATGAGCAGGCATAGCCGGAACCGCCCTCAGCCTGCTCATCCGCGCAGAACTCGGTCAACCCGGAACC

Smag_02 TTATTCTCAACCAACCACAAAGATATCGGCACCCTTTACCTAATCTTCGGCGCATGAGCAGGCATAGCCGGAACCGCCCTCAGCCTGCTCATCCGCGCAGAACTCGGTCAACCCGGAACC

Smag_05 TTATTCTCAACCAACCACAAAGATATCGGCACCCTTTACCTAATCTTCGGCGCATGAGCAGGCATAGCCGGAACCGCCCTCAGCCTGCTCATCCGCGCAGAACTCGGTCAACCCGGAACC

Smag_06 TTATTCTCAACCAACCACAAAGATATCGGCACCCTTTACCTAATCTTCGGCGCATGAGCAGGCATAGCCGGAACCGCCCTCAGCCTGCTCATCCGCGCAGAACTCGGTCAACCCGGAACC

Smag_07 TTATTCTCAACCAACCACAAAGATATCGGCACCCTTTACCTAATCTTCGGCGCATGAGCAGGCATAGCCGGAACCGCCCTCAGCCTGCTCATCCGCGCAGAACTCGGTCAACCCGGAACC

Smen_01 TTATTCTCAACCAACCACAAAGACATTGGCACCCTTTACCTAATCTTCGGCGCATGAGCAGGCATAGCCGGAACCGCTCTCAGCCTGCTCATCCGCGCAGAACTCGGTCAACCCGGAACC

Smag_01 CTCCTAGGAGACGACCAGATCTACAATGTAATTGTTACCGCCCATGCCTTCGTAATAATCTTCTTCATAGTAATACCTATCATAATCGGAGGATTTGGAAACTGACTAGTCCCACTTATA

Smag_02 CTCCTAGGAGACGACCAGATCTACAATGTAATTGTTACCGCCCATGCCTTCGTAATAATCTTCTTCATAGTAATACCCATCATAATCGGAGGATTTGGAAACTGACTAGTCCCACTTATA

Smag_05 CTCCTAGGAGACGACCAGATCTACAATGTAATTGTTACCGCCCATGCCTTCGTAATAATCTTCTTCATAGTAATACCCATCATAATCGGAGGATTTGGAAACTGACTAGTCCCACTTATA

Smag_06 CTCCTAGGAGACGACCAGATCTACAATGTAATTGTTACCGCCCATGCCTTCGTAATAATCTTCTTCATAGTAATACCTATCATAATCGGAGGATTTGGAAACTGACTAGTCCCACTTATA

Smag_07 CTCCTAGGAGACGACCAGATCTACAATGTAATTGTTACCGCCCATGCCTTCGTAATAATCTTCTTCATGGTAATACCCATCATAATCGGAGGATTTGGAAACTGACTAGTCCCACTTATA

Smen_01 CTCCTAGGAGATGACCAGATCTACAATGTAATTGTCACCGCCCATGCCTTCGTAATAATCTTCTTCATAGTAATACCTATTATAATCGGAGGATTTGGAAACTGACTAGTTCCACTTATA

Smag_01 ATCGGCGCCCCCGACATAGCATTTCCCCGCATAAATAACATAAGCTTTTGACTACTACCTCCCTCCTTCCTACTCCTACTAGCCTCCTCCACAGTAGAAGCAGGAGCCGGCACAGGATGA

Smag_02 ATCGGCGCCCCCGACATAGCATTTCCCCGCATAAATAACATAAGCTTTTGACTACTACCTCCCTCCTTCCTACTCCTACTAGCCTCCTCCACAGTAGAAGCAGGAGCCGGCACAGGATGA

Smag_05 ATCGGCGCCCCCGACATAGCATTCCCCCGCATAAATAACATAAGCTTTTGACTACTACCTCCCTCCTTCCTACTCCTACTAGCCTCCTCCACAGTAGAAGCAGGAGCCGGCACAGGATGA

Smag_06 ATCGGCGCCCCCGACATAGCATTTCCCCGCATAAATAACATAAGCTTTTGACTACTACCTCCCTCCTTCCTACTCCTACTAGCCTCCTCCACAGTAGAAGCAGGAGCCGGCACAGGATGA

Smag_07 ATCGGCGCCCCCGACATAGCATTTCCCCGCATAAATAACATAAGCTTTTGACTACTACCTCCCTCCTTCCTACTCCTACTAGCCTCCTCCACAGTAGAAGCAGGAGCCGGCACAGGATGA

Smen_01 ATCGGCGCCCCCGACATAGCATTTCCCCGCATAAATAACATAAGCTTTTGACTACTACCTCCCTCCTTCCTACTCCTACTAGCCTCCTCCACAGTAGAAGCAGGAGCCGGCACAGGATGA

Smag_01 ACCGTATACCCACCATTAGCAGGCAACCTAGCCCATGCCGGCGCATCAGTAGACCTAGCCATTTTTTCACTCCACCTAGCAGGAATCTCCTCCATCCTAGGAGCAATCAACTTCATCACC

Smag_02 ACCGTATACCCACCATTAGCAGGCAACCTAGCCCATGCCGGCGCATCAGTAGACCTAGCCATTTTTTCACTCCACCTAGCAGGAATCTCCTCCATCCTAGGAGCAATCAACTTCATCACC

Smag_05 ACCGTATACCCACCATTAGCAGGCAACCTAGCCCATGCCGGCGCATCAGTAGACCTAGCCATTTTTTCACTCCACCTAGCAGGAATCTCCTCCATCCTAGGAGCAATCAACTTCATCACC

Smag_06 ACCGTATACCCACCATTAGCAGGCAACCTAGCCCATGCCGGCGCATCAGTAGACCTAGCCATTTTTTCACTCCACTTAGCAGGAATCTCCTCCATCCTAGGAGCAATCAACTTCATCACC

Smag_07 ACCGTATACCCACCATTAGCAGGCAACCTAGCCCATGCCGGCGCATCAGTAGACCTAGCCATTTTTTCACTCCACCTAGCAGGAATCTCCTCCATCCTAGGAGCAATCAACTTCATCACC

Smen_01 ACCGTATACCCACCGTTAGCAGGCAACCTAGCCCATGCCGGCGCATCAGTAGACCTAGCCATTTTTTCACTCCATCTAGCAGGAATCTCCTCCATCCTAGGAGCAATCAACTTCATCACC

Smag_01 ACCGCCACTAACATAAAACCCCCAGCCCTATCACAATACCAAACCCCCCTGTTCGTATGATCCGTCCTTATCACAGCTGTCCTCCTACTACTCTCACTTCCCGTACTTGCTGCCGGCATC

Smag_02 ACCGCCACTAACATAAAACCCCCAGCCCTATCACAATACCAAACCCCCCTGTTCGTATGATCCGTCCTTATCACAGCTGTCCTCCTACTACTCTCACTTCCCGTACTTGCTGCCGGCATC

Smag_05 ACCGCCACTAACATAAAACCCCCAGCCCTATCACAATACCAAACCCCCCTGTTCGTATGATCCGTCCTTATCACAGCTGTCCTCCTACTACTCTCACTTCCCGTACTTGCTGCCGGCATC

Smag_06 ACCGCCACTAACATAAAACCCCCAGCCCTATCACAATACCAAACCCCCCTGTTCGTATGATCCGTCCTTATCACAGCTGTCCTCCTACTACTCTCACTTCCCGTACTTGCTGCCGGCATC

Smag_07 ACCGCCACTAACATAAAACCCCCAGCCCTATCACAATACCAAACCCCCCTGTTCGTATGATCCGTCCTTATCACAGCTGTCCTCCTACTACTCTCACTTCCCGTACTTGCTGCCGGCATC

Smen_01 ACCGCCACTAACATAAAACCCCCAGCCCTATCACAATACCAAACCCCCCTGTTCGTATGATCTGTCCTTATCACAGCTGTCCTCCTACTACTCTCACTTCCCGTACTTGCCGCTGGCATC

<

Smag_01 ACCATGCTACTAACAGACCGAAACCTAAACACCACCTTCTTCGATCCAGCTGGAGGGGGAGACCCAATCCTATACCAGCACCTCTTC

Smag_02 ACCATGCTACTAACAGACCGAAACCTAAACACCACCTTCTTCGATCCAGCTGGAGGGGGAGACCCAATCCTATACCAGCACCTCTTC

Smag_05 ACCATGCTACTAACAGACCGAAACCTAAACACCACCTTCTTCGATCCAGCTGGAGGGGGAGACCCAATCCTATACCAGCACCTCTTC

Smag_06 ACCATGCTACTAACAGACCGAAACCTAAACACCACCTTCTTCGATCCAGCTGGAGGGGGAGACCCAATCCTATACCAGCACCTCTTC

Smag_07 ACCATGCTACTAACAGACCGAAACCTAAACACCACCTTCTTCGATCCAGCTGGAGGGGGAGACCCAATCCTATACCAGCACCTCTTC

Smen_01 ACTATGCTACTAACAGACCGAAACCTAAACACCACCTTCTTCGATCCAGCTGGAGGGGGAGACCCAATCCTATACCAGCACCTCTTC
