# Supplementary material for: The role of demographic history and selection in shaping genetic diversity of the Galápagos penguin (Spheniscus mendiculus)
Source: PLoS One. 2020 Jan 7;15(1):e0226439. doi: 10.1371/journal.pone.0226439 (PMC6946592; doi:10.1371/journal.pone.0226439)
Supplement: S1 Table — (DOCX) [file pone.0226439.s003.docx]

**S1 Table**

**Allele frequencies observed at the five microsatellite loci (B3-2, G3-6, G2-2, M1-11, and H2-6) assessed for the Magellanic and Galápagos penguins. Allele calling refer to the size (in base pairs) of PCR amplification product.**

| Species | B3-2 | Frequency | G3-6 | Frequency | G2-2 | Frequency | M1-11 | Frequency | H2-6 | Frequency |
| --- | --- | --- | --- | --- | --- | --- | --- | --- | --- | --- |
|  |  |  |  |  |  |  |  |  |  |  |
| Magellanic | 297 | 0.100 | 262 | 0.077 | 373 | 0.423 | 130 | 1.000 | 286 | 0.865 |
| penguin | 299 | 0.280 | 268 | 0.077 | 375 | 0.019 |  |  | 290 | 0.135 |
|  | 301 | 0.540 | 270 | 0.115 | 379 | 0.019 |  |  |  |  |
|  | 303 | 0.060 | 272 | 0.115 | 381 | 0.481 |  |  |  |  |
|  | 307 | 0.020 | 274 | 0.038 | 383 | 0.038 |  |  |  |  |
|  |  |  | 276 | 0.077 | 387 | 0.019 |  |  |  |  |
|  |  |  | 280 | 0.019 |  |  |  |  |  |  |
|  |  |  | 282 | 0.038 |  |  |  |  |  |  |
|  |  |  | 284 | 0.077 |  |  |  |  |  |  |
|  |  |  | 286 | 0.038 |  |  |  |  |  |  |
|  |  |  | 288 | 0.058 |  |  |  |  |  |  |
|  |  |  | 290 | 0.019 |  |  |  |  |  |  |
|  |  |  | 296 | 0.077 |  |  |  |  |  |  |
|  |  |  | 298 | 0.019 |  |  |  |  |  |  |
|  |  |  | 300 | 0.096 |  |  |  |  |  |  |
|  |  |  | 302 | 0.058 |  |  |  |  |  |  |
| Galápagos | 297 | 1.000 | 266 | 0.882 | 383 | 1.000 | 130 | 1.000 | 286 | 1.000 |
| penguin |  |  | 268 | 0.118 |  |  |  |  |  |  |
